# Redefining Normal: Cytokine Dysregulation in Long COVID and the Post-Pandemic Healthy Donors

**DOI:** 10.3390/ijms26178432

**Published:** 2025-08-29

**Authors:** Zoia R. Korobova, Natalia A. Arsentieva, Natalia E. Lyubimova, Areg A. Totolian

**Affiliations:** 1 Laboratory of Molecular Immunology, Saint Petersburg Pasteur Institute, Mira St., 14, 197101 St. Petersburg, Russia; 2 Faculty of Medicine, First Pavlov State Medical University of St. Petersburg, L’va Tolstogo St. 6-8, 197022 St. Petersburg, Russia

**Keywords:** COVID-19, pandemic, long COVID, cytokines, healthy donors, recovered individuals

## Abstract

The COVID-19 pandemic has caused over 7 million deaths, but its legacy extends beyond mortality. SARS-CoV-2 infection induces immune alterations that persist post-recovery, manifesting not only in long COVID (LC) but also in healthy individuals. Cytokines serve as critical orchestrators of these processes. The goal of this study is to investigate post-pandemic immune remodeling through cytokine assessment in both patients with LC and healthy donor, and to compare the post-pandemic population with pre-pandemic controls to find changes in the immune responses and cytokine profiles. A panel of 47 immune mediators (cytokines, chemokines, and growth factors) was measured with the MAGPIX multiplex analysis. LC was characterized by an increase in IL-7, IL-8, IL-17F, IL-18, EGF, FGF-2, PDGF-AA, sCD40L, and MCP-3 and a decrease in IL-4, IL-13, IL-22, IL-27, and FLT-3L. Comparing post-pandemic recovered individuals with pre-pandemic healthy cohort, we saw an upregulation of IL-13 and MCP-3 and a downregulation of MDC, M-CSF, IL-12, and IL-17F. While LC is characterized by persistent immune imbalance—particularly in cytokine networks—our data emphasize the critical need to study healthy donors in both pre- and post-pandemic eras when analyzing and interpreting these changes.

## 1. Introduction

The COVID-19 pandemic has caused over 7 million deaths globally [1], but its legacy extends beyond mortality. A significant proportion of survivors develop long COVID (LC), a multisystem condition affecting 10–30% of non-hospitalized and 50–70% of hospitalized patients. LC manifests as debilitating symptoms including fatigue, cognitive dysfunction (“brain fog”), dyspnea, and immune dysregulation, persisting for months to years after initial infection. Notably, LC frequently occurs after mild acute COVID-19, suggesting that even a non-severe course of infection can trigger long-term immunological consequences [2,3,4].

SARS-CoV-2 infection induces profound immune system alterations that persist post-recovery. Acute disease severity correlates with CD4+/CD8+ T-cell lymphopenia, elevated exhaustion markers (PD-1, TIM-3), and cytokine storm (hyperinflammation driven by IL-6, TNF-α, IFN-γ) that promotes tissue damage [5]. Crucially, these disruptions often outlast viral clearance: exhausted T cells, reduced effector memory populations, and interferon signatures (IFN-β/λ) persist for ≥8 months [6], while naive T-cell depletion and thymic output deficits (reflected by low TREC levels) suggest impaired immune reconstitution [7]. Such dysregulation may explain LC’s heterogeneous symptoms and increased susceptibility to reinfection/autoimmunity.

Cytokines—key mediator molecules of immunity—serve as critical orchestrators of these processes. They regulate T-cell differentiation (e.g., Th1/Th2/Th17 polarization), B-cell activation, and inflammatory cascades. In acute COVID-19, cytokine imbalances (e.g., elevated IL-6, IL-1β) predict progression to severe disease or a lethal outcome [8,9]. However, their role in the recovery process after the infection remains poorly defined. Persistent subclinical inflammation (so-called “smoldering cytokine activation”) is hypothesized to sustain LC pathology—driving neuroinflammation, microthrombosis, and autonomic dysfunction—yet comprehensive cytokine profiling across the recovery process is lacking.

The goal of this study is to investigate post-pandemic immune remodeling through systematic cytokine assessment in both patients with LC and healthy donors. This research compares the post-pandemic population with pre-pandemic controls to find the most prominent changes in the immune responses and cytokine profiles.

## 2. Results

### 2.1. Baseline Cohorts’ Characteristics

For the first step of this study, we assessed clinical and laboratory markers to establish the baseline characteristics of our patient cohorts. We specifically focused on three distinct groups: patients with long COVID, as well as pre-pandemic and post-pandemic cohorts (Table 1). This initial step allowed us to identify differences and similarities among these groups, providing a foundation for further analysis of the long-term effects of COVID-19 and the pandemic in general.

### 2.2. Measurements of Cytokines in Long COVID and Post- and Pre-Pandemic Donors

**Figure 1:**
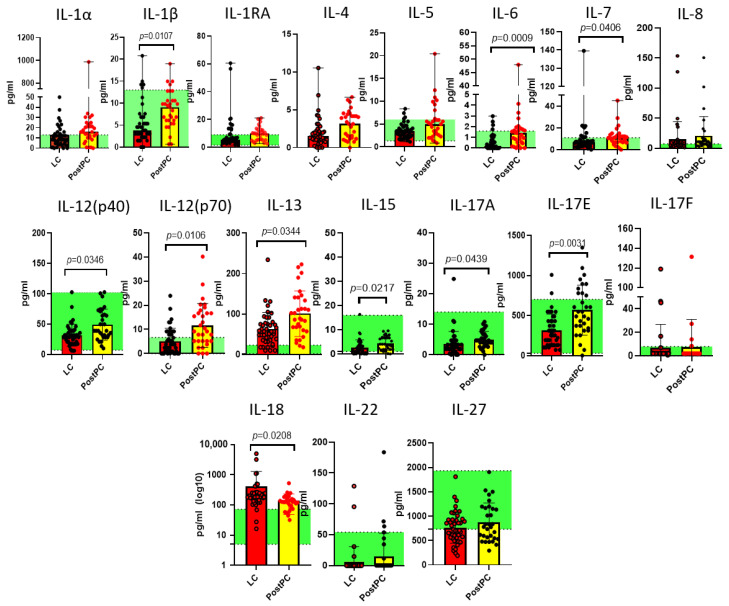
Interleukins demonstrating statistically significant differences between the long COVID (LC, n = 44) cohort and post-pandemic recovered individuals (PostPC) and pre-pandemic controls (Q25–Q75) presented as a green stripe, n = 33: IL-1α, IL-1β, IL-1RA, IL-4, IL-5, IL-6, IL-7, IL-8, IL-12(p40), IL-12(p70), IL-13, IL-15, IL-17A, IL-17E, IL-17F, IL-18, IL-22, and IL-27. Concentrations are presented in pg/mL. Red dots represent statistically significant differences from the pre-pandemic controls.

**Figure 2:**
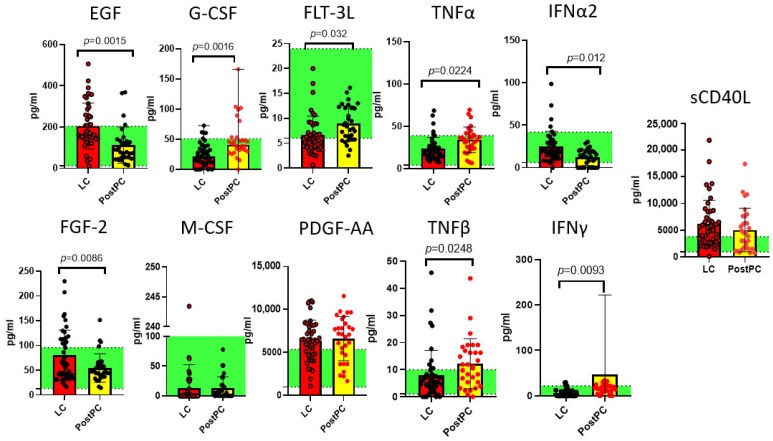
Growth factors, tumor necrosis factors, interferons, and other soluble ligands had statistically significant differences between the long COVID (LC, n = 44) cohort and post-pandemic recovered individuals (PostPC) and pre-pandemic controls (Q25–Q75) presented as a green stripe, n = 33: Epidermal growth factor (EGF), fibroblast growth factor 2 (FGF-2), FMS-like tyrosine kinase 3 ligand (FLT-3L), granulocyte colony-stimulating factor (G-CSF), macrophage colony-stimulating factor (M-CSF), platelet-derived growth factor AA (PDGF-AA), tumor necrosis factors α and β (TNFα, TNFβ), interferons α and γ (IFNα, IFNγ), and soluble CD40 ligand (sCD40L). Concentrations are presented in pg/mL. Red dots represent statistically significant differences from the pre-pandemic controls.

**Figure 3:**
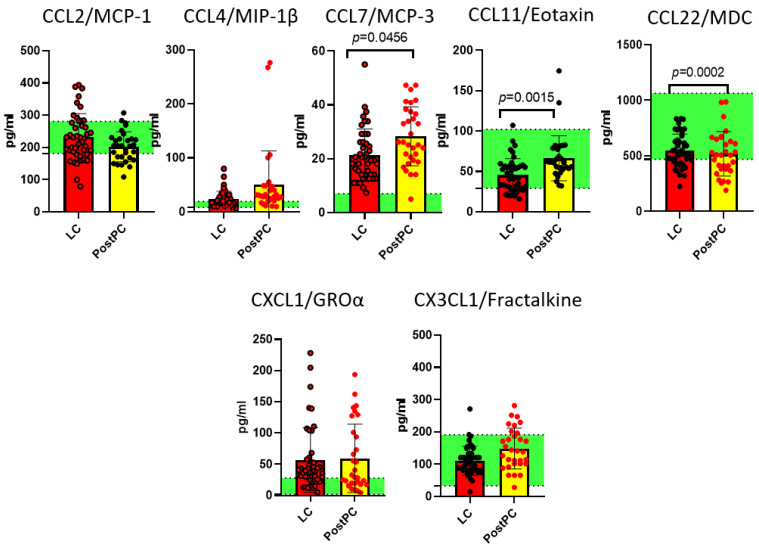
Chemokines demonstrating statistically significant differences between the long COVID (LC, n = 44) cohort and post-pandemic recovered individuals (PostPC) and pre-pandemic controls (Q25–Q75) presented as green stripe, n = 33: CCL2/MCP-1, CCL4/MIP-1β, CCL7/MCP-3, CCL11/Eotaxin, CCL22/MDC, CXCL1/GROα, and CX3CL1/Fractalkine. Concentrations are presented in pg/mL. Red dots represent statistically significant differences from the pre-pandemic controls.

Results demonstrating statistically significant differences are presented in Figure 1, Figure 2 and Figure 3.

As demonstrated in Figure 1, Figure 2 and Figure 3, our analysis revealed statistically significant differences in 36 out of 47 analytes across study groups, including cytokines (e.g., IL-1β, IL-6, TNFα), growth factors (e.g.,G-CSF, M-CSF, PDGF-AA), and chemokines (e.g., CCL2/MCP-1, CCL22/MDC).

LC was characterized by an increase in IL-7, IL-8, IL-17F, IL-18, EGF, FGF-2, PDGF-AA, sCD40L, MCP-3 and a decrease in IL-4, IL-13, IL-22, IL-27, and FLT-3L. These findings reflect a profound immune imbalance in LC, marked by dysregulated Th1/Th17-driven inflammation (↑ IL-17F, IL-18) overriding protective Th2 responses (↓ IL-4, IL-13). We note a defective cytotoxicity and immune surveillance (↓ FLT-3L) and failed tissue repair (↑ pro-fibrotic EGF/FGF-2, ↓ IL-22-mediated mucosal protection).

For post-pandemic recovered individuals, changes in cytokine profile remained: IL-7, IL-8, Il-14, IL-17F, IL-18, and FLT-3L were different from pre-pandemic controls, keeping a low-grade inflammation profile via IL-8/IL-17F/IL-18 and impairment in viral defenses. However, do these persistent cytokine shifts represent an adaptive ‘immune memory’—enhancing future defense—or do they reflect chronic impairment that could undermine long-term health, even in successfully recovered individuals?

### 2.3. Comparison of Pre-Pandemic Healthy Controls and Post-Pandemic Recovered Individuals

When comparing concentrations between cohorts, we observed unexpected contrast between pre-pandemic healthy donors and post-pandemic recovered individuals, suggesting that the immunological landscape may have shifted even in individuals without documented SARS-CoV-2 infection. This prompted us to investigate these changes. We decided to systematically evaluate changes in circulating cytokine profiles between donors sampled before and after the COVID-19 pandemic.

To quantify and visualize these differences, we adapted the volcano plot—a tool traditionally used in transcriptomics to identify differentially expressed genes—to analyze cytokine concentration changes. Volcano plots display two key metrics: fold change (FC), the ratio of post-pandemic to pre-pandemic median cytokine concentrations (or vice versa). An FC > 1 indicates higher post-pandemic levels, while FC < 1 reflects depletion.

Statistical significance (−log10(*p*-value)) reflected the strength of evidence against the null hypothesis (no difference between post- and pre-pandemic eras).

**Figure 4:**
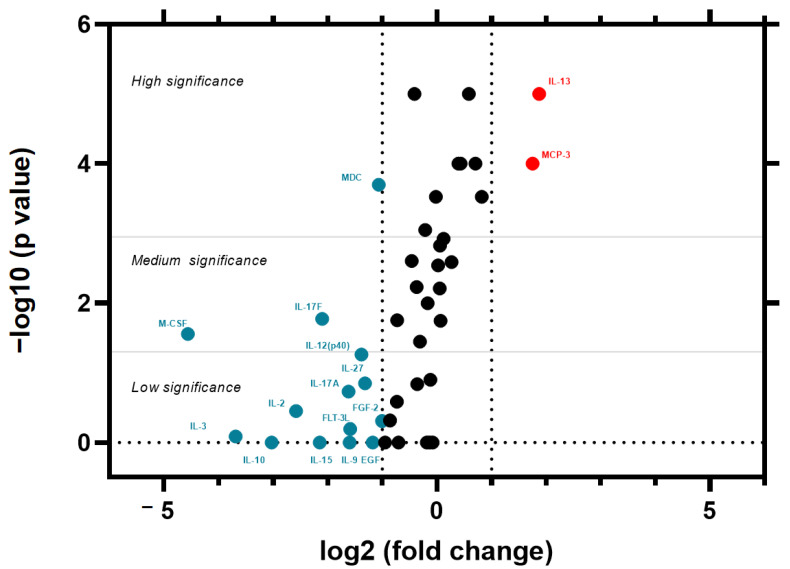
Differential cytokine regulation (concentration dynamics) was evaluated using a volcano plot comparing healthy donors sampled before (n = 30) and after (n = 33) the COVID-19 pandemic. *X*-axis fold change (FC): Log_2_-transformed ratios of medians, *Y*-axis statistical significance: −log_10_(*p*-values) from Kruskal–Wallis. Turquoise dots—downregulated cytokine; red dots—upregulated cytokines; black dots—cytokines not demonstrating dynamic changes. Thresholds for differential regulation were defined as FC magnitude: |log_2_(FC)| ≥ 1 (indicating ≥2-fold change). Significance tiers: low: −log_10_(*p*) ≥ 1.301 (*p* ≤ 0.05), medium: 1.3 < −log_10_(*p*) ≤ 2.95 (0.00111 ≤ *p* < 0.05), high: −log_10_(*p*) > 2.95 (*p* < 0.00111). Thresholds were composed of Bonferroni correction for multiple comparisons. The pronounced upward shift in IL-13 and MCP-3 alongside suppression of dendritic cell chemoattractants, i.e., MDC, suggests pandemic-associated immune reprogramming.

The analysis revealed alterations in the post-pandemic cohort (Figure 4): significant upregulation of pro-inflammatory mediators IL-13 and MCP-3, suggesting a Th2-biased immune response. We also noted marked downregulation of key immunomodulators, including dendritic cell chemoattractant MDC, macrophage colony-stimulating factor M-CSF, Th1/Th17-polarizing cytokines IL-12 and IL-17F, and other markers with less significance. Notably, Bonferroni correction (α = 0.0011) confirmed changes in IL-13, MCP-3, and MDC, while other cytokines exhibited lesser significance (*p* < 0.05).

## 3. Discussion

SARS-CoV-2 infection, regardless of initial severity (mild or severe), often triggers a hyper-inflammatory state marked by significantly elevated levels of multiple cytokines, such as IL-6, IL-8, and TNF-α [10]. While an initial proinflammatory cytokine surge is a standard antiviral defense, SARS-CoV-2 possesses specific viral proteins that effectively suppress the critical early type I interferon (IFN-I) response [11]. This evasion disrupts the normal immune orchestration, frequently leading to a prolonged, dysregulated, and often excessive cytokine response during the acute phase.

This cytokine dysregulation does not always resolve with the acute infection. Studies focusing on long COVID reveal a persistent and abnormal inflammatory signature. A notable finding is an abnormally diffuse inflammatory cytokine profile detectable in long COVID patients for at least 8 months post-infection, a pattern distinct from individuals who recovered asymptomatically [6]. As cytokines primarily act in a localized, paracrine, or autocrine manner within tissues, their pathogenic effects in long COVID might occur despite levels in the peripheral blood not always being elevated [12]. The detection of these cytokines in blood, therefore, likely reflects spillover from ongoing localized inflammatory processes rather than being the primary site of action.

Our study revealed that several markers were changed in patients with long COVID when compared to healthy pre-pandemic controls and post-pandemic recovered individuals: EGF, FLT3L, IL-13, IL-18, MCP-3, and MIP-1β. EGF—epidermal growth factor—is a marker of fibrosis in acute COVID. Studies show that EGF and TGF-β1 expression increases with COVID-19 severity, while their serum levels paradoxically decrease in critical cases. This might suggest a shift from systemic circulation to localized tissue activity, potentially one behind fibrosis formation [13]. FLT3L (FMS-like Tyrosine Kinase 3 Ligand) demonstrated correlations with recovery post-infections in children [14].

Earlier we demonstrated lowered FLT3L levels in convalescent patients in 1–3 months after recovery [15]. This cytokine is one of the key hemopoetic factors involved in dendritic cell growth [16]. FLT3L serves as an indicator of myelopoiesis and provides a negative correlation with leukocytes in aplastic anemia and neutropenia [17]. As FLT3L levels remain lowered in both patients with LC and without it, it is possible to speculate on an existing shift in hemopoesis in the post-pandemic era.

IL-13 is elevated in severe COVID-19 patients requiring mechanical ventilation and is linked to poor outcomes. It promotes lung pathology via hyaluronan (HA) deposition, contributing to respiratory failure, and drives fibrotic pathways and chronic inflammation, potentially contributing to persistent pulmonary and metabolic dysfunction in long COVID [18]. IL-13 is a canonical Th2 cytokine that binds a receptor shared with IL-4 (IL-13Rα1/IL-4Rα). It drives pathological processes in severe COVID-19, including mucus production, collagen deposition, and IgE class-switching [19].

IL-18 is a member of the IL-1 cytokine family; IL-18 is a critical mediator of innate and adaptive immunity, produced by monocytes, macrophages, and dendritic cells. Its key function is to potently stimulate IFN-γ production, frequently in synergy with IL-12 or IL-15. This synergy is crucial for driving potent Th1 responses and activating cytotoxic CD8 T and NK cells, positioning IL-18 as a master regulator of inflammatory cascades [20]. IL-18 remains elevated for months post-infection and correlates with cardiovascular complications, including endothelial dysfunction and thromboinflammation [21]. In long COVID, IL-18 is linked to neuroinflammation and autoimmunity, potentially driving fatigue and cognitive symptoms (“brain fog”) [22]. It is one of the so-called ‘constant’ markers of infection we described in our previous studies [23].

MIP-1β is part of the hyperinflammatory response in severe COVID-19, promoting macrophage and T-cell recruitment to infected tissues [24]. It is elevated in ICU patients and correlates with lung injury and multiorgan failure. Persistent MIP-1β may drive chronic immune activation and microvascular damage, contributing to long COVID symptoms like myalgia and dysautonomia [25].

An important factor influencing the development of long COVID is the SARS-CoV-2 variant during initial infection, which not only shapes the cytokine and chemokine profile [25,26] but also determines the clinical presentation of long COVID [27,28]. Notably, the Omicron variant appears to induce classic long COVID symptoms—such as fatigue, post-exertional malaise, myalgia, dyspnea, paresthesias, chest pain, and orthostatic intolerance—despite the absence of persistent peripheral cytokine elevation [29]. Further study of long COVID patients may be of interest with consideration of the viral variant.

When compared to influenza, COVID-19 demonstrated 71% higher rates of secondary infection [30]. Long COVID patients exhibit T-cell exhaustion (high PD-1 expression) and dysregulated B-cell maturation, resembling chronic viral infections like HIV [31].

Studies show that even mild COVID-19 can lead to a significant decline in T and B cells (key components of adaptive immunity) up to 10 months post-infection, suggesting long-term immune suppression or exhaustion. Research suggests SARS-CoV-2 may damage bone marrow function, reducing the output of new immune cells. This could explain the prolonged depletion of lymphocytes (T/B cells) and monocytes observed in recovered patients [32].

COVID-19 can trigger autoantibodies targeting interferons (IFNs) and other immune components, which may contribute to long COVID symptoms like fatigue and neuroinflammation [33]. Some patients develop antibodies against ACE2 and persistence of spike protein immune cells, potentially disrupting vascular and neurological function [34].

Maciel et al., in their 2024 study, highlight a disruption of cytokine homeostasis in individuals 2 years after COVID-19 [35]. Discordance between peripheral blood and cerebrospinal fluid (CSF) cytokines hints at localized CNS inflammation [36].

Our study reveals a paradigm-shifting observation: healthy donor samples taken after the COVID-19 pandemic exhibit different immune profiles compared to their pre-pandemic counterparts. This divergence may suggest that the pandemic’s legacy extends far beyond acute infection or long COVID (LC). One interpretation of our data is an immunological reset—likely driven by viral exposure, vaccination, altered environmental exposures (e.g., reduced pathogen circulation during lockdowns), or even psychosocial stressors.

We identified significant dysregulation in key immunomodulators—notably elevated IL-13 and MCP-3, alongside depleted MDC (CCL22)—that persists even in healthy individuals without long COVID. These findings extend our prior work demonstrating MDC depletion as a hallmark of COVID-19 convalescence (both acute and 3 months post-infection) and may suggest new immunological characteristics in the post-pandemic population [37]. While the functional implications are not yet fully understood, such shifts could potentially enhance defense against reinfection but might also theoretically predispose individuals to fibrotic disorders (given the role of IL-13) or chronic inflammatory states (via MCP-3-mediated monocyte activation). In long COVID patients, a failure to resolve these altered immune signatures could contribute to the persistence of symptoms.

Our data, though requiring caution in terms of interpretation, raise a methodological conundrum: Which cohort truly represents a “control” in post-pandemic studies? One interpretation of our data is that pre-pandemic donors may reflect a state of “naïve” immunity, though their direct relevance to the current, pandemic-altered immune landscape could be limited. While post-pandemic recovered individuals might demonstrate real-world baseline shifts, their profiles likely represent factors beyond infection alone. Therefore, we tentatively propose that dual-baseline designs, which compare long COVID cohorts against both pre- and post-pandemic healthy donors, could help quantify pandemic-associated immune shifts.

For studies aiming to define a “healthy” post-pandemic cohort, we suggest that future work prioritize the direct comparison of vaccinated versus unvaccinated individuals, ideally using samples from both before and after the pandemic where possible. Such a design would be highly valuable for disentangling the specific immunological effects of vaccination from those of natural infection, and we further recommend stratifying participants by detailed vaccination status and infection history.

## 4. Materials and Methods

### 4.1. Study Cohorts and Ethical Considerations

This cross-sectional study included the following three cohorts:

- Long COVID (LC) Group (*n* = 44): Individuals meeting WHO criteria for diagnosis, with persistent psychoneurological symptoms (e.g., cognitive impairment via Montreal Cognitive Assessment; anxiety/depression via Hospital Anxiety and Depression Scale) and somatic complaints (fatigue, dyspnea) lasting >12 weeks post-acute infection. All participants had PCR-confirmed prior SARS-CoV-2 infection.
- Post-Pandemic Recovered Individuals (*n* = 33): Asymptomatic individuals with confirmed prior mild COVID-19 (PCR-positive) and full recovery >1 year before sampling.
- Pre-Pandemic Naïve Controls (*n* = 30): Healthy donors sampled in early 2020 (pre-COVID-19 pandemic), confirmed SARS-CoV-2-naïve via nucleocapsid IgG seronegativity.

Full information on demographics and clinical characteristics of these patients is presented in Table 1 in Section 2.

Ethical approval was obtained from the Saint Petersburg Pasteur Institute Ethics Committee (Protocols #3, 22.11.2016, #67 28.04.2021, and #84, 16.02.2023). All participants provided written informed consent.

### 4.2. Blood Collection and Plasma Isolation

Peripheral blood (5 mL) was collected in K3 EDTA tubes (VACUETTE^®^, Griener Bio-One, Kremsmünster, Austria) prior to therapeutic interventions. Plasma was isolated via centrifugation at 300× *g* for 7 min at 25 °C. Aliquots were stored at −80 °C until cytokine analysis.

### 4.3. Cytokine Assessment

A panel of 47 immune mediators (cytokines, chemokines, and growth factors) was quantified using the MILLIPLEX^®^ MAP Human Cytokine/Chemokine Magnetic Bead Panel (MilliporeSigma, Cat. # HCYTOMAG-60K, Burlington, MA, USA) on the Luminex^®^ MAGPIX^TM^ (Luminex, Austin, TX, USA) platform. Analytes included interleukins and selected pro-inflammatory cytokines (IL-1α, IL-1β, IL-2, IL-3, IL-4, IL-5, IL-6, IL-7, IL-9, IL-12(p40), IL-12(p70), IL-13, IL-15, IL-17A/CTLA8, IL-17-E/IL-25, IL-17F, IL-18, IL-22, IL-27, IFNα2, IFNγ, TNFα, TNFβ/Lymphotoxin-α[LTA]); chemokines (CCL2/MCP-1, CCL3/MIP-1α, CCL4/MIP-1β, CCL7/MCP-3, CCL11/Eotaxin, CCL22/MDC, CXCL1/GROα, CXCL8/IL-8, CXCL9/MIG, CXCL10/IP-10, CX3CL1/Fractalkine); anti-inflammatory cytokines (IL-1Ra, IL-10); growth factors (EGF, FGF-2/FGF-basic, Flt-3 Ligand, G-CSF, M-CSF, GM-CSF, PDGFAA, PDGFAB/BB, TGFα, VEGF-A); and sCD40L. Samples were processed per manufacturer protocol: 25 μL plasma was incubated with antibody-conjugated beads overnight (4 °C), followed by detection with biotinylated secondary antibodies and streptavidin-PE.

Several steps were taken to minimize technical and pre-analytical variability. All blood samples were taken into the same type of EDTA tubes and processed immediately under a standardized protocol for aliquoting. All aliquots were subsequently stored long-term in the same −80 °C freezer to ensure uniform storage conditions. Furthermore, the entire sample processing and experimental workflow were conducted by the same core team of personnel to minimize human factors. To ensure quality control, all samples were analyzed in duplicate.

### 4.4. Statistical Analysis

Data normality was assessed using the Kolmogorov–Smirnov test. Non-normally distributed cytokine levels were compared across groups with the Kruskal–Wallis test, followed by Dunn’s test for pairwise comparisons. Differential cytokine expression was evaluated using volcano plots (*X*-axis–fold change (FC): Log_2_-transformed ratios, Y Statistical significance: −log_10_(*p*-values) from Kruskal–Wallis. Thresholds for differential expression were defined as FC magnitude: |log_2_(FC)| ≥ 1 (indicating ≥2-fold change). Significance tiers: Low: −log_10_(*p*) ≥ 1.301 (*p* ≤ 0.05), medium: 1.3 < −log_10_(*p*) ≤ 2.95 (0.00111 ≤ *p* < 0.05), high: −log_10_(*p*) > 2.95 (*p* < 0.00111). Thresholds were composed of Bonferroni correction for multiple comparisons. Analyses were performed using GraphPad Prism 8.0 (Dotmatics, Boston, MA, USA), Microsoft Excel 2013 (Microsoft Corporation, Redmond, WA, USA), and RStudio 2025.05.1.

## 5. Conclusions

Our findings suggest that the COVID-19 pandemic may have altered cytokine-based immune responses beyond the well-documented cytokine disruptions in acute COVID-19 and smoldering cytokine activation in long COVID patients. While long COVID is characterized by persistent immune imbalance—particularly in cytokine networks—our data emphasize the critical need to study healthy donors in both pre- and post-pandemic eras when analyzing and interpreting these changes. The current immune landscape implies that even successfully recovered individuals and infection-naïve populations now exist in an immunologically altered environment shaped by viral exposures, vaccination campaigns, and behavioral adaptations. This paradigm necessitates dual-cohort studies to distinguish true long COVID pathology from broader pandemic-related immune shifts.

## Figures and Tables

**Table 1:** Demographics and health status of cohorts included in the study.

Group	Long COVID	Post-Pandemic Recovered Individuals	Pre-Pandemic Naïve Controls
*n*	44	33	30
Age (Me, Q25–Q75)	38, 29–48	40, 35–48	44, 38–52
Gender distribution (% female/% male)	72.7/27.3	60.1/0.39	80/20
Anti-SARS-CoV-2 IgG	+ (Positive)	+ (Positive)	− (Negative)
COVID-19 severity	84.1% mild, 15.9% moderate/severe	100% Mild	n/a
Vaccination status	40.9% vaccinated with Sputnik V	100% vaccinated with Sputnik V	n/a
Health status and existing comorbidities	100% hypertension, 56.8% hair loss, 52.3% poor appetite, 45.5% nonspecific abdominal pain. 75% had comorbidities: gastrointestinal (31.8%), renal/urinary (18.2%), cardiovascular disorders (6.8%); 22.7% BMI > 25 kg/m^2^.	No residual symptoms or active comorbidities reported.	No residual symptoms or active comorbidities reported.

## Data Availability

The data presented in this study are available from the corresponding author upon request due to institutional privacy policy.

## Abbreviations

The following abbreviations are used in this manuscript:

FC	Fold Change
G-CSF	Granulocyte Colony-Stimulating Factor
GM-CSF	Granulocyte-Macrophage Colony-Stimulating Factor
IFN	Interferon
IL	Interleukin
IP-10	Interferon Gamma-Induced Protein 10
LC	Long COVID
MCP	Monocyte Chemoattractant Protein
M-CSF	Macrophage Colony-Stimulating Factor
MDC	Macrophage-Derived Chemokine
MIP	Macrophage Inflammatory Protein
PDGF	Platelet-Derived Growth Factor
PostPC	Post-Pandemic Recovered Individuals
PrePC	Pre-Pandemic Controls
TGF	Transforming Growth Factor
Th	T Helper
TNF	Tumor Necrosis Factor
VEGF	Vascular Endothelial Growth Factor
